# Investigation of the Effect of NaCl Concentrations on the Formation of Amyloid Fibrils During the Cooking of Wheat Noodles

**DOI:** 10.3390/foods14162892

**Published:** 2025-08-20

**Authors:** Ying Liang, Chunlei Zheng, Liu Yang, Minqian Huang, Jiajia Liu, Hao Liu, Baoshan He, Jinshui Wang

**Affiliations:** 1 College of Biological Engineering, Henan University of Technology, Zhengzhou 450001, China; yliang@haut.edu.cn (Y.L.); zcl128@stu.haut.edu.cn (C.Z.); yangliu6@stu.haut.edu.cn (L.Y.); huangminqian@stu.haut.edu.cn (M.H.); ljj922@stu.haut.edu.cn (J.L.); liuhao0051@gmail.com (H.L.); 2 College of Food Science and Engineering, Henan University of Technology, Zhengzhou 450001, China; hebaoshan@126.com

**Keywords:** amyloid fibrils, gluten, sodium chloride, thermal processing, wheat noodles

## Abstract

In our previous study, we observed that sodium chloride (NaCl) influences the formation of amyloid fibrils (AFs) by gluten in cooked wheat noodles. However, the underlying mechanisms of NaCl’s effect on AF formation during the cooking process remain unclear. This study systematically investigates the impact of NaCl concentration (0–2.0%, *w*/*w*) and cooking time (0–7 min) on AF formation. ThT fluorescence and Congo red confirmed AF formation across all NaCl concentration levels. At low NaCl concentrations, Na^+^/Cl^−^ shielding reduced electrostatic repulsion, enabling ordered β-sheet stacking, yielding long fibrils (1193 nm) with high β-sheet content (41.5%), dense cross-β structures, and elevated hydrophobicity (H_0_ = 9980). Stable zeta potential and gradual particle growth (376 to 1193 nm) supported controlled elongation. Conversely, high NaCl concentrations disrupted hydrogen bonding, forming shorter fibrils (820 nm) with reduced β-sheets (28.9%) and lower hydrophobicity (H_0_ = 5923). Rapid ThT kinetics (*df/dt* = 77,535 FU/min) and SE-HPLC profiles suggest that elevated concentrations of NaCl inhibit AF formation while inducing the generation of amorphous aggregates. These findings clarify the balance between ionic shielding and hydrophobic interactions in AF assembly, offering strategies to optimize noodle texture. Future studies should address the digestibility and health implications of salt-modulated AFs for functional food applications.

## 1. Introduction

Amyloid fibrils (AFs), protein aggregates characterized by their canonical cross-β-sheet architecture, exhibit significant potential in food colloid stabilization and functional material development due to their high aspect ratios, abundant surface functional groups, and unique interfacial properties [1,2]. Emerging evidence indicates that sodium chloride (NaCl) critically modulates AF formation pathways by altering protein intermolecular interactions. For instance, in β-lactoglobulin systems, 50–100 mM NaCl accelerates fibril nucleation through electrostatic screening, ultimately forming rigid β-sheet-enriched architectures [3]. In contrast, NaCl in rice glutelin and flaxseed protein systems facilitates directional β-sheet assembly by inducing specific peptide hydrolysis (particularly <10 kDa hydrophobic fragments) [4,5]. Furthermore, soy protein isolate forms 8 nm diameter flexible fibrils under the influence of 80 mM NaCl, significantly enhancing emulsion stability [6], while kidney bean protein constructs high-modulus gel networks through the cross-linking of linear aggregates under high-salt conditions (300 mM NaCl) [7]. Although existing studies have preliminarily elucidated NaCl-mediated molecular mechanisms of amyloid fibril formation in ex vivo protein models, the multicomponent interactions within complex food matrices may induce significant divergence in its regulatory behaviors. Current investigations predominantly lack integration with practical food systems and their processing protocols.

As a pivotal processing adjunct in noodle production, NaCl plays a crucial role in modulating dough properties [8,9,10]. NaCl enhances the storage modulus (G’), loss modulus (G″) and extensibility of dough by promoting intermolecular interactions among gluten proteins, which leads to the formation of a more compact gluten network structure. Under low-concentration conditions or short cooking durations, this additive can effectively maintain the hardness and elasticity of noodles [11]. However, excessive NaCl addition may induce partial disruption of the gluten network, which promotes starch leaching during cooking and consequently elevates the cooking loss rate [12]. While the impact of NaCl on specific sensory attributes (e.g., elasticity) remains debated, it is universally recognized to fulfill an irreplaceable function in optimizing dough processing properties, enhancing noodle structural integrity, and coordinating flavor profiles [9,13]. Intriguingly, recent studies reveal that gluten undergoes amyloid-like amyloid fibril formation during thermal processing [14], with NaCl shown to induce the fibrillar restructuring of gluten networks in cooked noodles [15]. This implies that NaCl may modulate noodle quality through regulating AF formation pathways; however, the dynamic regulation mechanisms underlying AF assembly in thermal food processing systems remain mechanistically elusive.

Our preliminary work demonstrated NaCl-induced AF formation in cooked wheat noodles [15]. We hypothesize that NaCl concentration differentially modulates fibril morphology through ionic shielding and hydrophobic interactions. To unravel the dynamic regulation of AFs by NaCl during food processing, this study uses a wheat noodle model to examine the effects of NaCl concentrations (0–2.0%) and cooking time (0–7 min) through multi-scale characterization, including Thioflavin T (ThT) fluorescence kinetics, Congo red binding assays, Fourier-transform infrared (FTIR) spectroscopy, and atomic force microscopy (AFM). Our results elucidate the mechanisms of the salt-dependent formation of amyloid fibrils, provide theoretical insights for the precise control of protein structure in flour processing, and allow us to promote the application of functional AFs in food technology. However, the mechanism has not yet been elucidated.

## 2. Materials and Methods

### 2.1. Materials

The materials used included the following: Family Banquet Wheat Flour (COFCO International, Beijing, China); Thioflavin T (2390-54-7, 97%, Shanghai Maclin Biochemical Technology Co., Ltd., Shanghai, China); Congo red indicator (Tianjin Kemi Ou Chemical Reagent Co., Ltd., Tianjin, China); 8-anilino-1-naphthalenesulfonic acid (ANS, ≥96%, Shanghai Maclin Biochemical Technology Co., Ltd.); sodium chloride (NaCl) and sodium hydroxide (Tianjin Tianli Chemical Reagent Co., Ltd., Tianjin, China). All reagents were of analytical grade.

### 2.2. Preparation of Noodles

The noodle preparation method we used was adapted from Liang et al. [16]. For the blank group, 100 g of wheat flour and water was mixed at a ratio of 1:0.4 (*w*/*w*). The resulting dough was sheeted and cut into noodles, which were heated in a water bath at 100 °C with stirring at 300 g/min for 0 min, 1 min, 3 min, 5 min, and 7 min to obtain wheat gluten. For the experimental groups, NaCl was added at concentrations (x%) of 0.4%, 1.6%, or 2.0% (*w*/*w*). The composition of the remaining components was identical to that of the blank group.

### 2.3. Extraction of AFs

A slightly modified version of the research method of Lambrecht et al. [17] was used. Amounts of of 1.5 g of lyophilized noodle powder with 0%, 0.4%, 0.8%, 1.2%, 1.6% and 2.0% NaCl and 1 mg of α-amylase (2900 U/10 mL; Shanghai Macklin Biochemical Technology Co., Ltd., Shanghai, China) were placed into 50 mL centrifuge tubes numbered 1–4, 10 mL of 0.02% sodium azide solution was added, and then the mixture was shaken for 24 h at 37 °C and centrifuged (10,000× *g*, 10 min) to remove the supernatant. Then, 1 mg proteinase K (400 U; Shanghai Macklin Biochemical Technology Co., Ltd., Shanghai, China), 10 mg trypsin 2500 U (Shanghai Macklin Biochemical Technology Co., Ltd., Shanghai, China), and 10 mL sodium azide solution were added to the centrifuge tube, which was placed in a shaker and shaken at 37 °C for 48 h. After removal, it was placed in an ice-water bath for 5 min; centrifugation commenced (10,000× *g*, 10 min), and then we removed the supernatant, SF1. To the precipitate, we added 8 mL of (0.05 M, pH 7.0) phosphate-buffered solution. We shook this (25 °C, 150 × *g*) for 16 h, centrifuged it (8000× *g*, 10 min), obtained the supernatant, SF2, took the supernatant, SF2, lyophilized and ground the powder, passed it through a 100 mesh sieve, and put it into a desiccator for storage.

### 2.4. Thioflavin T Fluorescence (ThT)

Following the method of Loveday et al. [18] with minor modifications, a 3.0 mM ThT stock solution was formulated in phosphate–NaCl buffer (0.01 M phosphate, 150 mM NaCl, pH 7.0). This stock solution was filter-sterilized using a 0.22 μm PES membrane (Jinteng, Zhoushan, China) and stored at 4 °C in brown glass bottles. For the working solution, the stock was diluted 50-fold in the same phosphate–NaCl buffer, yielding a final ThT concentration of 60 μM. Fluorescence measurements involved adding 50 μL of the sample to 3 mL of working solution. After brief vortexing and 1 min incubation at room temperature, fluorescence was recorded using a Multimode microplate reader (Spark, Tecan Group Ltd., Männedorf, Switzerland) at excitation and emission wavelengths of 440 nm and 482 nm, respectively.

ThT fluorescence data were fitted with the Boltzmann constant in Equation (1), which characterizes the fluorescence intensity as a function of cooking time. The maximum rate of increase in fluorescence (*df/dt*)_max_ was calculated from Equation (2), where f_t_ is the fluorescence at time t, A_2_ and A_1_ denote the maximum and minimum fluorescence, respectively, t_1/2_ is the time when the fluorescence reaches the midpoint, and t_c_ is the time constant.

(1) $$ft=A2•\frac{A1-A2}{1+\exp\left(\frac{t-t\frac{1}{2}}{tc}\right)}$$

(2) $$\left(\frac{df}{dt}\right)=\frac{\left(A2-A1\right)}{4tc}$$

### 2.5. Congo Red Staining Observation

Congo red staining analysis experiments were carried out on the formed AFs using a UV-visible spectrophotometer (model A590; Shanghai Aoyi Instrument Co., Ltd., Shanghai, China) [15]. Congo red solution at a concentration of 0.5 mM was prepared using 50 mM phosphate solution (pH 7.0). For the staining analysis, the sample was dissolved in Congo red solution to prepare a 10 mg/mL sample–Congo red mixture. The concentrated protein solution (10 μL) was spotted onto a slide and air-dried. The dried sample was then covered with 20–30 μL of staining solution (comprising 80% ethanol, excess NaCl, excess Congo red, and 0.05% sodium azide, stirred and filtered prior to use). After brief incubation, excess stain was carefully removed by blotting it with filter paper without disturbing the sample. The slide was subsequently air-dried completely at room temperature. Stained samples were examined using a polarized light microscope (50ipol, Nikon Corporation, Tokyo, Japan). Congo red solution alone served as the control. Fluorescence excitation and emission wavelengths were set at 400 nm and 700 nm, respectively.

### 2.6. Size Exclusion High-Performance Liquid Chromatography (SE-HPLC)

The molecular weight distribution of AFs was detected using an LC system (LC-2010, Shimadzu, Kyoto, Japan) equipped with a BioSep SEC-S4000 column (5 µm, 300 mm × 7.8 mm; Phenomenex, Torrance, CA, USA) [17]. The SE-HPLC analysis protocol involved the following steps: Protein (1.0 mg) was dissolved in 1 mL of phosphate buffer (0.05 M, pH 6.8) with SDS (2.0% *w*/*v*) and agitated at room temperature for 60 min. After centrifugation (10,000× *g*, 10 min), the supernatant was filtered through 0.45 μm PES filters (Tianjin Jinteng, China). Chromatographic separation involved utilizing a BioSep SECS4000 column (5 μm, 300 mm × 7.8 mm, Phenomenex, Torrance, CA, USA). The mobile phase, consisting of acetonitrile/water (1:1, *v*/*v*) with 0.1% (*v*/*v*) trifluoroacetic acid, was delivered at 1 mL/min. Eluted components were detected by UV absorbance at 214 nm.

### 2.7. Fourier Transform Infrared (FTIR) Spectrum

AF secondary structures in noodle were analyzed using Fourier transform infrared spectroscopy (FT-IR, Tensor II spectrometer, Nicolet 67, Thermo Nicolet, Madison, WI, USA) [19]. Samples (5 mg) mixed with KBr (500 mg) were thoroughly ground and pressed into transparent pellets. Spectra were acquired across the full spectral range (400–4000 cm^−1^) with 64 scans. The amide I region (1600–1700 cm^−1^) underwent spectral deconvolution and second-derivative analysis using PeakFit software (v4.12; Systat Software, Inc., San Jose, CA, USA). Gaussian curve fitting enabled the quantification of secondary structure components based on relative areas: aggregated strands (1600–1624 cm^−1^), β-sheet (1624–1640 cm^−1^), random coil (1640–1650 cm^−1^), α-helix (1650–1660 cm^−1^), β-turn (1660–1685 cm^−1^), and antiparallel β-sheet (1685–1700 cm^−1^).

### 2.8. Endogenous Fluorescent Light

The fluorescence properties of dough gluten proteins were analyzed by the method of Han et al. [20]. A lyophilized glutenin sample (50 mg) was weighed and dissolved in 10 mL of 0.05 mol/L acetic acid solution (AAS). The sample was then shaken at room temperature for 2 h (250 g/min) and centrifuged (10,000× *g*, 15 min) to collect the supernatant. The supernatant was finally diluted to 1 mg/mL with the above AAS under the following conditions (fluorescence spectrophotometer (Model F-7100, Hitachi High-Tech Corporation, Tokyo, Japan)): an excitation wavelength of 280 nm; an emission wavelength of 290–410 nm; and a slit width of 5 nm.

### 2.9. Particle Size and ζ-Potential Analysis

The particle size was measured according to a known procedure [21]. The AF samples were diluted to 2 mg/mL, and added into the nanoparticle sizer (NANOS90, Malvern Panalytical Ltd., Malvern**,** UK) and ζ potential analyzer (Zetasizer Nano Z, Malvern Panalytical Ltd., UK) successively to record the average particle size and ζ potential value of the samples.

### 2.10. Surface Hydrophobicity (H_0_)

Surface hydrophobicity (H_0_) was quantified in triplicate via the fluorescent probe 1-aniline-8-naphthalenesulfonate (ANS) following established protocols [17]. Protein samples were serially diluted in 0.01 M sodium phosphate buffer (pH 7.0) to concentrations ranging from 0.05 to 0.50 mg/mL. For each dilution, 200 μL aliquots were transferred to a black 96-well microplate (Greiner BioOne, Kremsmünster, Austria) and mixed with 10 μL of ANS solution (8.0 mM). Fluorescence measurements were conducted immediately using a multifunctional microplate reader (Spark, Tecan, Männedorf, Switzerland), with excitation at 390 nm and emission detection at 480 nm. The relative fluorescence intensity (RFI) was derived by subtracting the fluorescence intensity of the ANS solution alone from that of the protein–ANS mixture. The surface hydrophobicity (H_0_) was then defined as the slope of the linear regression plot of RFI versus protein concentration.

### 2.11. Atomic Force Microscope (AFM)

The molecular morphology of AFs was characterized using an AFM (Jupiter XR, Oxford Instruments, Abingdon, UK) operating in AC mode [22]. For imaging, protein samples were diluted to approximately 0.01 µg/mL in 0.05 M acetic acid. A 10 µL aliquot of this solution was deposited onto a freshly cleaved mica substrate, air-dried rapidly (3–5 min), and subsequently imaged by AFM.

### 2.12. Statistical Analysis

All experiments were performed in triplicate, and data are presented as mean values. Statistical analysis was conducted using IBM SPSS Statistics 27 (IBM Corp., Armonk, NY, USA). Significant differences among experimental groups were evaluated by one-way analysis of variance (ANOVA), followed by Tukey–Kramer honestly significant difference (HSD) post hoc tests, with a significance level set at *p* < 0.05. Data visualization was performed using Origin 2021.

## 3. Results and Discussion

### 3.1. Formation of AFs

#### 3.1.1. Analysis of ThT

**Figure 1:**
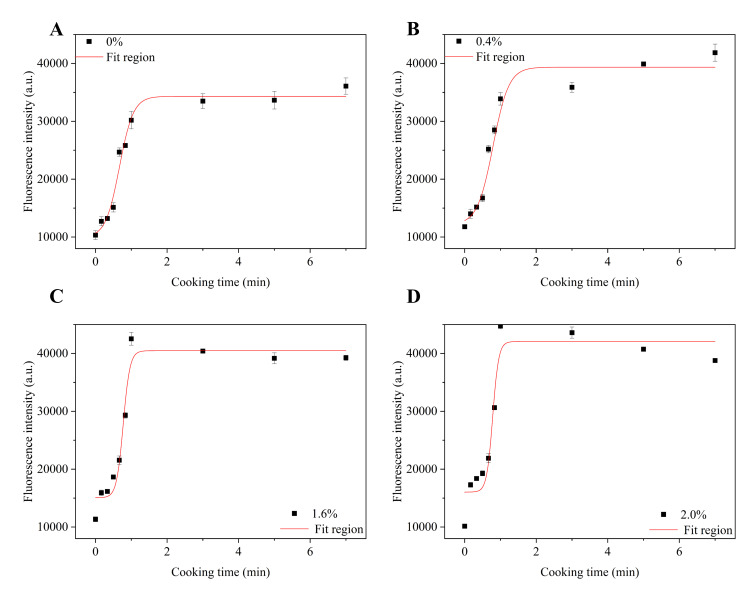
ThT fluorescence intensity at different NaCl concentrations. (**A**) ThT fluorescence intensity at 0% NaCl concentration, (**B**) ThT fluorescence intensity at 0.4% NaCl concentration, (**C**) ThT fluorescence intensity at 1.6% NaCl concentration, (**D**) ThT fluorescence intensity at 2.0% NaCl concentration.

The ThT fluorescence assay, given that ThT specifically binds to β-sheet structures in AFs, has been extensively employed to monitor amyloidogenesis [23]. Boltzmann equation-fitted sigmoidal kinetics demonstrate that AF formation during wheat noodle cooking progresses through three distinct phases, a lag phase, growth phase, and plateau phase [24], with these stages characterized by the maximum growth rate (*df*/*dt*) and half-completion time (*t*_1/2_) of protofibril aggregation [25]. As shown in Figure 1A–D, the lag phase exhibited a concentration-dependent extension with elevated NaCl concentration levels across all samples. Subsequently, the ThT fluorescence intensity underwent rapid escalation during the growth phase before stabilizing at the plateau phase. This kinetic pattern aligns with observations by Li et al. [26], who reported that analogous ThT signal amplification in rice glutelin fibrillization under thermal stress ultimately reached a plateau phase. This phenomenon likely suggests that rapid hydrolysis and nucleation during the initial heating phase generate abundant reactive monomers and β-sheet-enriched protofibrils, thereby driving the accelerated elevation of ThT fluorescence intensity through enhanced β-sheet stacking density [27,28]. Low NaCl concentrations reduce electrostatic repulsion (e.g., between glutamic acid residues), enhancing ordered β-sheet stacking and fibril elongation [5]. Notably, the peak ThT fluorescence intensity was observed upon 1 min heating with a 2.0% NaCl concentration, which may have been the result of the salt-promoted formation of short protofibrils that amplified ThT-binding sites via exposed β-sheet edges [29]. The AFs incubated in 2.0% NaCl exhibited a lower ThT fluorescence intensity compared to other samples. This observation may be attributed to the neutralization of protein surface charges by salt ions, which reduces intermolecular repulsive forces. Consequently, this process promotes the formation of amorphous aggregates rather than well-ordered fibrils, leading to a decrease in β-sheet structures available for ThT binding [29,30]. Furthermore, electrostatic screening was observed to enhance the lateral aggregation of short protofibrils while suppressing their longitudinal elongation with the structural order. This dual effect resulted in the formation of a dense fibrillar network accompanied by a reduced β-sheet structural order. Consequently, the number of accessible ThT-binding sites was significantly diminished [31]. The distinct ThT fluorescence patterns (Figure 1) and kinetic parameters (Table A1) indicate salt-dependent amyloid fibril formation pathways. These trends are directly corroborated by quantitative AFM morphological analysis (Section 3.4 ), revealing that low NaCl (0.4%) yields elongated fibrils (1193 nm), while high NaCl (2.0%) produces shortened aggregates (820 nm). Consistent with our findings, Li, Wang, Zhang, Yu and Chen [5] observed enhanced fibril formation with increasing ionic concentrations. They attributed this phenomenon to accelerated aggregation kinetics under elevated ionic strengths, where charge screening effects neutralize positively charged residues and attenuate intermolecular repulsive forces. However, at excessively high ionic concentrations, a prolonged time may be required to complete protofibril assembly. Parallel evidence emerged from Zhang, Tang, Wen, Yang, Li and Deng [7], who demonstrated that progressive ionic strength augmentation thermodynamically favors the hierarchical assembly of kidney bean fibrils through salt-bridge stabilization mechanisms.

Table A1 lists the kinetic parameters of primary amyloid fibril formation. After incubation with 0–2.0% NaCl, the growth rate of AFs (*df*/*dt*) increased from 31,325.25 ± 8359.19 FU min^−1^ to 77,535.67 ± 40,442.19 FU min^−1^. The electrostatic screening effects of Na^+^ and Cl^−^ accelerate protofibril aggregation by reducing electrostatic repulsion through the neutralization of the protein’s net surface charge. However, the half-life (t_1/2_) of AFs increased from 0.669 min to 0.783 min after salt incubation. A potential explanation is that salt addition increases protofibril yield, which requires more time to reach the plateau phase of fibril formation [26]. This may further suggest an increased energy barrier during the fibril maturation stage. Experimental findings from Feng et al. [32] reveal that β-sheet stability relies on the synergistic interplay of multiple forces—primarily governed by hydrogen bonding, with secondary contributions from hydrophobic interactions. Salt-induced weakening of hydrogen bonding could impose additional conformational constraints, thereby prolonging the time required for protofibrils to attain a stable conformational state.

#### 3.1.2. Congo Red-Polarized Light Microscope Observation

**Figure 2:**
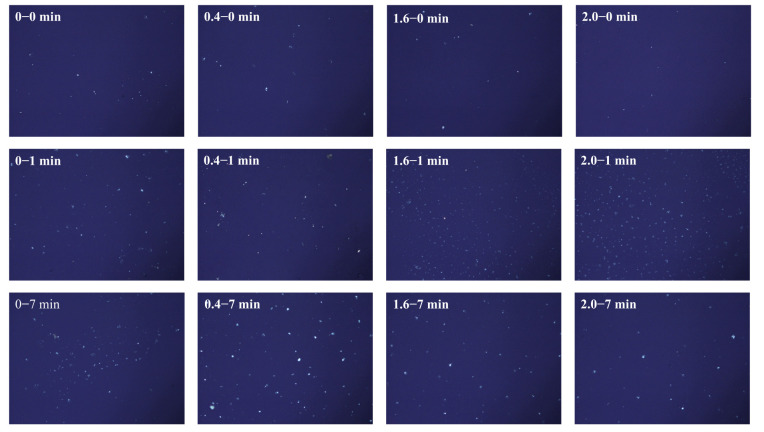
Effect of NaCl on the formation of the AF morphology of noodles during the cooking process.

The Congo red staining method is a diagnostic assay that differentiates AFs from non-fibrillar components by exploiting the dye’s preferential binding to cross-β conformations characteristic of amyloidogenic structures while exhibiting minimal affinity for non-β-sheet assemblies [33]. Upon binding, AFs exhibit characteristic apple-green birefringence under polarized light, serving as a hallmark of cross-β structural organization [34]. As shown in Figure 2, both NaCl concentration and cooking duration critically influence the formation and structural stability of AFs. Notably, compared to the absence of NaCl, systems with low NaCl concentrations demonstrated maintained signal stability over time, suggesting that moderate-charge screening enables the stepwise ordered stacking of β-sheet layers along the thermodynamically controlled pathway, ultimately yielding compact fibrillar architectures [15,35]. This ionic shielding mechanism is directly corroborated by FTIR spectral deconvolution (Section 3.2.1, Table 1), which reveals a significant increase in β-sheet content from 29.8% (0% NaCl) to 41.5% (0.4% NaCl, 7 min) alongside reduced random coils. The 39% enhancement in ordered β-stacking quantitatively validates the Na^+^/Cl^−^-mediated suppression of electrostatic repulsion as a driver for fibril elongation. Moreover, Na^+^ may stabilize fibrillar architectures by neutralizing surface negative charges on β-sheets originating from acidic residues, thereby reducing inter-strand electrostatic repulsion and promoting β-sheet registry [36].

Under high NaCl concentrations, initial heating triggered the rapid accumulation of birefringence signals, whereas prolonged heating reduced the signal intensity in these samples. This behavior reflects the kinetic competition between nucleation and disordered aggregation. Elevated ionic concentration initially accelerates amyloid nucleation through electrostatic screening [37], but excessive charge shielding disrupts the ordered hydrogen bonding arrangement required for β-sheet stacking. Consequently, while high ionic strength promotes fibrillar aggregation, it may simultaneously compromise the structural stability of the resulting fibrils [38,39]. This aligns with ThT kinetics (Figure 1): NaCl enhances assembly via charge screening, but excess salt disrupts β-sheet order [40,41]. It has been demonstrated that proteins are more prone to disordered aggregation at high NaCl concentrations, resulting in lower β-sheet content [39].

### 3.2. Structural Characterization of Fibrils

#### 3.2.1. Secondary Structure

FTIR spectroscopy was used to elucidate the effects of ions on protein conformational changes and molecular interactions. The amide I region (1600–1700 cm^−1^) provided critical insights into secondary structural features [42]. FTIR spectroscopy analysis of the amide I region revealed significant NaCl-dependent variations in AF secondary structure, as quantified in Table 1. In the absence of NaCl, a disordered conformation predominated, characterized by low β-sheet content (29.8%) alongside higher random coil (13.6%) and α-helix (18.0%) proportions. Adding low-concentration NaCl (0.4%) significantly increased β-sheet content to 36.6% while decreasing that of random coils to 12.3%. However, further increasing the NaCl concentration to 2.0% reduced the β-sheet content to 29.2% and elevated that of random coils to 14.2%. This effect suggests that at low NaCl concentrations, ionic shielding reduces electrostatic repulsion, thereby promoting intermolecular interactions and β-sheet formation. This observation is consistent with the findings of Juárez et al., who reported that moderate salt concentrations promote effective intermolecular interactions conducive to β-sheet assembly [37]. Furthermore, the characteristic redshift observed in the FTIR spectra at elevated salt concentrations indicates tighter β-sheet packing. This spectral shift suggests that NaCl stabilizes the β-sheet structure by enhancing interprotein hydrogen bonding and hydrophobic interactions [6]. Conversely, the structural instability induced by high NaCl concentrations, manifested as reduced β-sheet content and increased random coil content, likely stems from the disruption of the hydrogen-bonding network essential for β-sheet stability, leading to unstable aggregates prone to depolymerization [29]. This is consistent with observations that excessive charge shielding compromises fibril structural integrity [39]. Furthermore, Cao et al. [43] demonstrated that Hofmeister ions modulate fibril formation by altering hydrogen bonding, hydrophobic, and electrostatic interactions, thereby supporting the observed NaCl-dependent structural transitions.

#### 3.2.2. Molecular Weight Distribution

**Figure 3:**
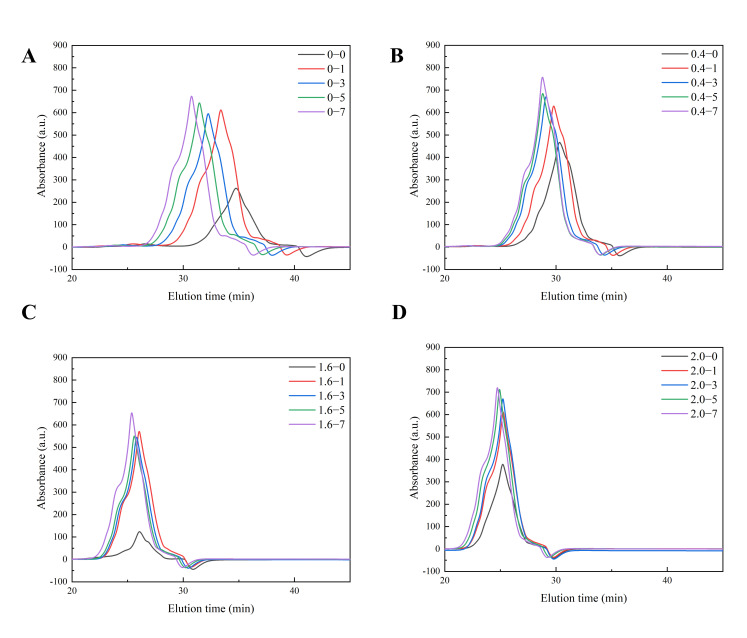
Effect of NaCl on the molecular weight of AFs formed from noodles during the cooking process: 0 min (black), 1 min (orange), 3 min (blue), 5 min (green), 7 min (purple). (**A**) Molecular weight distribution of AFs in noodles with 0% NaCl added, (**B**) Molecular weight distribution of AFs in noodles with 0.4% NaCl added, (**C**) Molecular weight distribution of AFs in noodles with 1.6% NaCl added and (**D**) Molecular weight distribution of AFs in noodles with 2.0% NaCl added.

SE-HPLC was used to characterize the aggregation states of AFs at different NaCl concentrations (Figure 3A–D). This chromatographic technique separates molecules based on their hydrodynamic size, using the elution time and peak area for both the qualitative and quantitative analysis of molecular composition [44]. Under all tested NaCl concentrations, the relative molecular weight of AFs exhibited a progressive increase with extended cooking duration. However, the concentration of NaCl played a critical role in determining the structural characteristics of the fibrils. In the absence of NaCl, the low-molecular-weight fragmentation peaks observed in SE-HPLC chromatograms likely correspond to monomers or oligomers not participating in fibril formation. These aggregates, predominantly adopting α-helical conformations, are incompatible with the β-sheet-ordered assembly required for AF formation [24]. The addition of low NaCl concentrations promotes the transition to a single high-molecular-weight peak, indicating enhanced fibril homogeneity. This phenomenon is attributed to ionic shielding effects: Na^+^ ions neutralize electrostatic repulsion between charged residues (e.g., glutamic acid) while strengthening hydrophobic interactions, thereby facilitating ordered β-sheet stacking and directional fibril elongation [15]. Notably, the smaller peptide fragments generated during partial hydrolysis may act as nucleation seeds that accelerate fibril growth [5]. This mechanism is supported by the ThT fluorescence stabilization and β-sheet enrichment detected through Fourier-transform infrared spectroscopy (Table 1, Figure 1). However, the addition of high NaCl concentrations induced peak broadening and a low-molecular-weight shift in chromatographic profiles, suggesting protein depolymerization or the formation of smaller aggregates. This phenomenon originates from high-ionic-strength-mediated electrostatic screening that disrupts the balance between hydrophobic interactions and electrostatic repulsion. Excessive Na^+^ ions shield surface charges, attenuating intermolecular electrostatic repulsion and allowing hydrophobic dominance, which may trigger nonspecific aggregation. Furthermore, elevated salt concentrations likely compromise β-sheet stability, not through polar residue (e.g., glutamine) repulsion, but by destabilizing the electrostatic/hydrogen bond networks essential for β-sheet maintenance [17,37,45]. Concurrently, salt competes with intraprotein hydrogen bonds, favoring random coil conformations over β-sheet structures. This is corroborated by the reduced β-sheet content in the FTIR spectra (Table 1) and fragmented morphologies observed via atomic force microscopy. These competing interactions drive the formation of amorphous aggregates rather than fibrillar structures [17,46].

#### 3.2.3. Tertiary Structure

**Figure 4:**
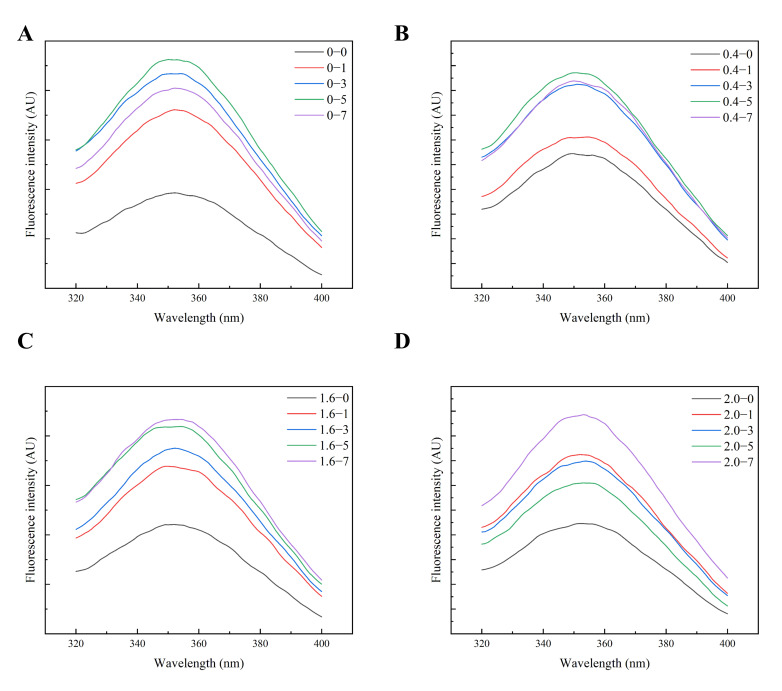
Effect of NaCl on the fluorescence properties of AFs formed from noodles during the cooking process: 0 min (black), 1 min (orange), 3 min (blue), 5 min (green), 7 min (purple). (**A**) Fluorescence properties of AFs formed in noodles with 0% NaCl added, (**B**) Fluorescence properties of AFs formed in noodles with 0.4% NaCl added, (**C**) Fluorescence properties of AFs formed in noodles with 1.6% NaCl added and (**D**) Fluorescence properties of AFs formed in noodles with 2.0% NaCl added.

Fluorescence spectroscopy was used to examine protein conformational changes and aggregation behavior [47]. Figure 4 illustrates the dynamic conformational shifts in AFs at different NaCl concentrations and under different heating durations. In the absence of NaCl, the fluorescence intensity exhibited unstable fluctuations during the heating process, showing an 80% increase at the 5 min mark followed by a 30% decrease. This behavior aligns with the findings reported by Yoshimura et al., who showed that, in glassy amorphous aggregates, the absence of ionic shielding allows dominant electrostatic repulsion to disrupt ordered β-sheet assembly. The initial fluorescence surge reflects transient hydrophobic exposure during thermal denaturation and acid hydrolysis, whereas the subsequent quenching originates from the burial of aromatic residues within disordered aggregates [38]. In contrast, samples with low NaCl concentrations exhibited a steady increase in fluorescence intensity, peaking at 7 min. This mirrors the observation by Song et al. that moderate ionic strength stabilizes hydrophobic interactions via charge screening, enabling the progressive alignment of β-sheet-rich fibrils [48]. The gluten fibrillization process is mediated by charge screening-stabilized hydrophobic interactions, facilitating the progressive alignment of β-sheet-rich protofilaments. The ThT fluorescence stabilization (Figure 1) and SE-HPLC sharp peaks (Figure 3) further confirm the synergistic role of ionic screening in balancing electrostatic repulsion and hydrophobic driving forces. High NaCl concentrations induced an initial surge in fluorescence intensity, followed by a sharp decline after 5 min. A plausible explanation is that NaCl reduces the dielectric constant of the solution, thereby enhancing hydrophobic interactions. This drives the protein conformation transition from a compact state to a more extended structure, exposing additional fluorophores and consequently increasing fluorescence intensity. However, at elevated salt concentrations, the strengthened intermolecular hydrophobic interactions promote the aggregation of polypeptide chains into dense fibrils or amorphous aggregates. The resultant burial of hydrophobic moieties within these aggregates prevents solvent accessibility, ultimately leading to fluorescence quenching [15,49]. The fluorescence quenching exhibited a direct correlation with the fibril fragmentation observed by AFM (Figure 6), a phenomenon mechanistically linked to the ion-mediated regulation of electrostatic screening, hydrophobic interactions, and hydrogen bonding networks. Consistent with the findings reported by Yu et al., elevated salt conditions destabilize fibril elongation stability by enhancing intermolecular repulsion between partially exposed charged residues (e.g., lysine), while concomitantly disrupting the structural architecture of AFs [24].

### 3.3. Physicochemical Properties of Fibrils

#### 3.3.1. ζ-Potential

**Figure 5:**
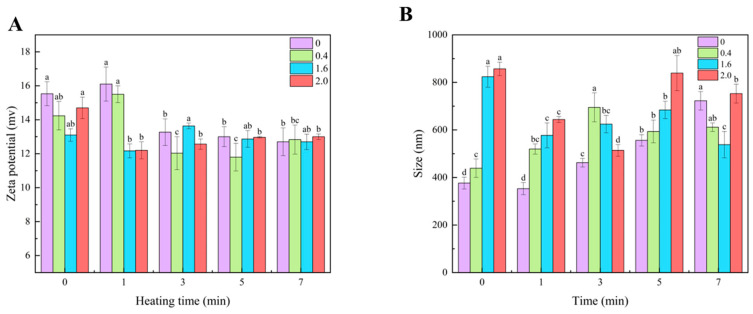
Relationship between NaCl concentration and zeta potential, and between NaCl concentration and average particle size during the cooking process. (**A**) Variation in ζ-potential of AFs with cooking time at different NaCl concentrations; (**B**) variation in average particle size of AFs with cooking time at different NaCl concentrations.

Zeta potential measurements were used to characterize the surface charge density and stability of proteins [39]. As depicted in Figure 5, the significantly higher zeta potential prior to NaCl addition demonstrates strong electrostatic repulsion between protein molecules. Subsequent introduction of NaCl resulted in a marked reduction in zeta potential, indicative of charge screening effects that attenuated interparticle repulsion, thereby facilitating amyloid fibril formation processes. This suggested that a reduction in the electrostatic repulsion up to a certain extent was beneficial for amyloid fibril formation. The initially elevated zeta potential reflects surface-exposed charged residues (e.g., lysine and glutamic acid) on the native protein conformation. Thermal denaturation-induced structural unfolding further exposes these ionizable groups, resulting in the transient amplification of surface charge density [5]. However, prolonged heating results in NaCl-mediated charge shielding. Low NaCl concentrations can partially neutralize and reduce the electrostatic repulsion forces without affecting hydrogen bonding, thereby enabling stable assembly of fibrils [6]. Elevated NaCl concentrations reduce interprotein electrostatic repulsion while enhancing hydrophobic interactions. The zeta potential decreased from 16.1 mV (0% NaCl) to 12.2 mV (2.0% NaCl) (Figure 5A), confirming that charge shielding attenuates electrostatic repulsion and promotes hydrophobic aggregation [26,50]. NaCl acts as a mediator influencing protein structure and function through mechanisms including charge environment modification and the promotion of fibril formation. A decrease in zeta potential indicates that ions neutralize the surface charges of proteins through an electrostatic shielding effect. Stronger electrostatic shielding enhances hydrophobic interactions, increasing gluten network density and potentially improving dough elasticity (G’) and shear resistance (e.g., enhanced noodle hardness).

#### 3.3.2. Average Particle Size

Average particle size, a sensitive and quantitative indicator of protein aggregation behavior, was used to evaluate trends in AF assembly [51]. Figure 5B depicts the particle size distribution of AFs in noodles after cooking at different NaCl concentrations. Compared to conditions in which NaCl was absent, heating with low NaCl concentrations induced particle size growth from 376 nm to 1193 nm, demonstrating that enhanced hydrophobic interactions via electrostatic screening drive the progressive elongation of AFs [43]. In contrast, samples with high NaCl concentrations exhibited limited particle size growth. This indicates that excessive ionic shielding disrupts the ordered amyloid fibril formation process. A previous study reported that significant changes in particle size are associated with the denaturation, unfolding, polypeptide disruption and assembly of protein during fibrillization [52]. The initial reduction in particle size under high NaCl concentrations during heating may be attributed to protein unfolding and the subsequent release of smaller peptides through acid hydrolysis, which likely act as nucleation seeds, a mechanism corroborated by SE-HPLC analysis (Figure 3). As the ionic concentration increased, the particle size of the formed fibers demonstrated a corresponding gradual enlargement post-treatment. Wang et al. observed that soybean protein particles initially exhibited size reduction, followed by a sharp increase in dimensions with prolonged heating duration [53]. Other researchers have also documented comparable phenomena, wherein non-heated rice glutelin particles undergo disintegration into smaller particulates during the initial heating phase, followed by the assembly of released peptides into larger fibrillar aggregates through prolonged thermal treatment [5,52]. Both Liu and Tang [54] and Zhang, Tang, Wen, Yang, Li and Deng [7] have reported analogous observations, demonstrating progressive enlargement in particle dimensions of vicilin proteins and kidney bean (*Phaseolus vulgaris* L.) particulates with incremental elevations in NaCl concentration. These findings demonstrate that NaCl-induced electrostatic shielding significantly enhances both aggregation propensity and fibril density, which aligns consistently with the ThT fluorescence quantification results.

#### 3.3.3. Surface Hydrophobicity

**Figure 6:**
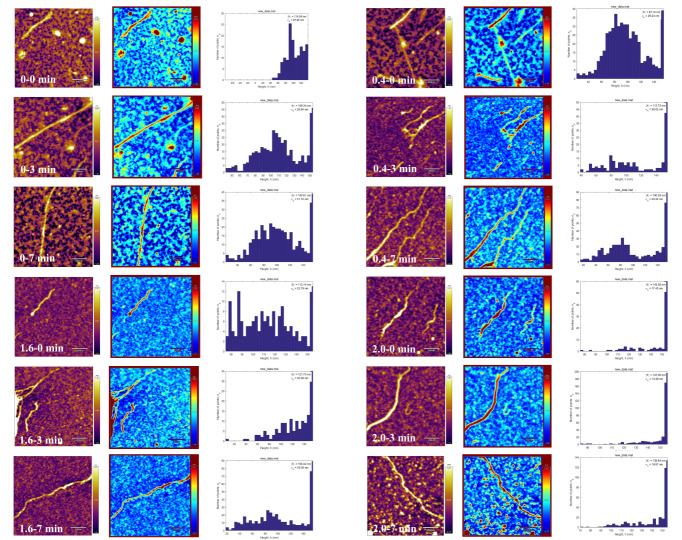
Effect of NaCl on the length and height distribution of AF profiles of noodle formation during the cooking process.

As a hydrophobic fluorescent dye, 8-Anilino-1-naphthalenesulfonic acid (ANS) is widely used to detect exposed hydrophobic groups [55]. H_0_ reflects changes in the tertiary structure during AF formation [56]. As shown in Table 2, compared to the absence of NaCl, the H_0_ value under low NaCl concentrations gradually increased with prolonged heating time, reaching a peak at 7 min. In contrast, samples under the influence of high NaCl concentrations attained their maximum H_0_ earlier on, followed by showing a subsequent decrease in this value. The higher H_0_ values observed under low NaCl concentrations correlate with stable ThT fluorescence (Figure 1) and ordered fibrils visualized by atomic force microscopy (Figure 6), collectively suggesting that low ionic strength promotes hydrophobicity-driven fibril assembly. This phenomenon may arise from moderate NaCl concentrations reducing electrostatic repulsions between charged residues (e.g., Asp/Glu), thereby progressively exposing the hydrophobic core (e.g., Phe) that is crucial for β-sheet stacking [39]. Exposed hydrophobic groups enhance interprotein contact, promoting amyloid fibril formation [57]. The H_0_ values observed under high NaCl concentrations were lower than those observed under low NaCl concentrations, indicating that hydrophobic interactions dominate protein self-assembly at elevated NaCl concentration levels. Previous studies have demonstrated that hydrophobic interactions between nonpolar groups facilitate β-sheet formation, while exposed aromatic amino acids promote fibril assembly [58]. According to zeta potential measurements, the balance between electrostatic repulsion and hydrophobic interactions at low NaCl concentrations facilitates fibril formation and elongation. Under high NaCl concentrations, however, the enhanced hydrophobic interactions between proteins due to electrostatic shielding disrupt the balance between hydrophobic forces and electrostatic repulsion, consequently impeding fibril growth and leading to the formation of irregular aggregates [39].

### 3.4. Morphological Analysis by Atomic Force Microscopy

AFM was used to observe the dynamic assembly of AF proteins during noodle cooking at different NaCl concentrations (Figure 6). In the absence of NaCl, although the primary fibrils exhibited progressive elongation from 529.2 nm to 1131.2 nm during heating, their height decreased from 114.6 nm to 100.6 nm within 7 min, demonstrating insufficient thermal stability. Under low NaCl concentrations, the fibrils initially exhibited shorter lengths (342.9 nm); however, they underwent rapid elongation to 1193.4 nm after 7 min of heating, ultimately forming elongated straight fibrils. Their height increased from 87.0 nm to 100.3 nm with narrowed distribution, indicating that low salt conditions promoted linear growth through lateral β-sheet cross-linking, thereby enhancing structural stability. Under high NaCl concentrations, AFM images revealed fragmentation in bright regions, likely resulting from salt-induced localized depolymerization, with fiber length subsequently increasing to 1159.2 nm after 7 min. Irregular and heterogeneous height distributions may induce fibril instability. Subsequent addition of 2.0% NaCl significantly inhibited fiber elongation (from 268.8 nm initially to 820.4 nm after 7 min), resulting in shortened, thickened fibrils. The reduced fibril height appears associated with diminished aggregation propensity or more loosely packed protein architectures [59].

The morphological divergence of AFs formed under high versus low NaCl concentrations can be rationalized through a fundamental polymer physics equation [60]:

(3) $$v=\frac{Rp}{Ri}$$

where v represents the kinetic chain length, R_p_ denotes the rate of fibril elongation, and R_i_ indicates the rate of fibril initiation. Under low NaCl concentrations, the diminished ThT fluorescence intensity compared to that under high NaCl concentrations indicates limited amyloid content in the sample, suggesting suppressed fibrillization kinetics. Equation (4) predicts that such conditions favor longitudinal growth, potentially yielding extended fibrils. At higher ionic strengths, the ratio of elongation to initiation rates decreases, indicating that the resulting amyloids will be shorter. It could be expected that for a higher initiation rate of amyloidogenesis, the amount of created fibrils will be large [29]. The direct application of Equation (4), while representing an obvious simplification, demonstrates excellent agreement with the experimental data. Under low NaCl concentrations, the equilibrium between electrostatic repulsion and hydrophobic interactions may facilitate the progressive stacking of β-sheets into AFs. This observation aligns with the sustained ThT fluorescence intensity (Figure 1), collectively confirming an ordered molecular assembly. These findings are consistent with established literature reports, which demonstrate that reduced ionic concentration enhances electrostatic repulsive forces, thereby accelerating fibril elongation processes [39]. The introduction of high NaCl concentrations induced excessive charge screening effects, which disrupted the hydrogen-bonding network and subsequently drove the formation of structurally unstable protein aggregates. The resultant fragmentation patterns correlate with both the SE-HPLC polydispersity index (Figure 3) and diminished β-sheet content (Table 1), which collectively align with salt-induced structural perturbation mechanisms [43]. Notably, under high NaCl concentrations, the height of AFs increased (139.8 nm) while their length decreased, suggesting lateral aggregation rather than longitudinal elongation. These results demonstrate that low NaCl concentrations promote ordered unidirectional fibril growth via electrostatic shielding, while high NaCl concentrations accelerate assembly but impede fibril elongation due to chaotic aggregation [5,37,61]. Similar behavior was also observed in hen egg white lysozyme and rice glutelin systems, where fibrils became shorter and fragmented when heated under high NaCl concentrations [5,29]. Previous studies have demonstrated that β-lactoglobulin, kidney bean protein isolate, rice bran globulin, and soy beta-conglycinin form longer entangled aggregates under elevated ionic strength conditions [7,50,62,63]. In contrast, the gluten fibrils in our study exhibited a pronounced propensity for fragmentation, highlighting the critical role of amino acid composition in salt sensitivity.

In conclusion, the significant structural differences in AFs formed under varying NaCl concentrations identified in this study provide a new molecular mechanistic perspective for understanding how salt regulates the macroscopic properties of noodles. The long and rigid AFs formed under low NaCl concentrations may incorporate more effectively into the gluten network. By enhancing network rigidity, they thereby contribute to improved noodle elasticity and chewiness, while reducing cooking loss. Conversely, the disordered aggregates generated under high NaCl concentrations may disrupt network coherence, leading to decreased stability in the formed network. This may partially explain the deterioration in noodle texture and increased cooking loss sometimes observed under high salt conditions. Therefore, precise control of salt concentration to guide the formation of AFs with desirable structures represents a promising strategy for optimizing the final quality of noodles.

## 4. Conclusions

The formation of AFs in gluten during noodle cooking was systematically modulated by NaCl concentration (0–2.0%) and heating duration (0–7 min). ThT fluorescence and Congo red birefringence both confirmed that AFs were generated under both low- and high-salt conditions. However, distinct structural and physicochemical properties emerged depending on NaCl concentration levels. At low NaCl concentrations, electrostatic shielding by Na^+^/Cl^−^ reduced intermolecular repulsion among charged residues (e.g., glutamate), enabling ordered β-sheet stacking. This produced elongated fibrils (1193.4 nm, AFM) with elevated β-sheet content (41.5%, FTIR) and enhanced hydrophobicity (*H*_0_ = 9980). Stable zeta potential and gradual size growth (376 → 1193 nm) indicated balanced electrostatic–hydrophobic interactions driving directional elongation. Controlled nucleation (t_1/2_ = 0.72 min) and moderate growth (*df*/*dt* = 35,171 FU min^−1^) aligned with stepwise β-sheet assembly. Conversely, high-NaCl-concentration-induced excessive charge screening disrupted hydrogen bonds, yielding shorter fibrils (820.4 nm, AFM), reduced β-sheet content (28.9%), and disordered aggregates (lower ThT fluorescence intensity). This shift towards disordered aggregation was further confirmed by SE-HPLC. Declined hydrophobicity (*H*_0_ = 5923) and zeta potential reflected hydrophobic burial in amorphous clusters. Rapid ThT kinetics (*df*/*dt*= 77,535 FU min^−1^, t_1/2_ = 0.78 min) favored amorphous aggregation over ordered fibrillization. Prolonged heating induced fibril fragmentation. In summary, low NaCl concentrations facilitate β-sheet formation and ordered assembly via electrostatic screening effects, whereas high NaCl concentrations destabilize β-sheet conformations and induce amorphous aggregate formation. These findings highlight the critical role of NaCl in balancing ionic and hydrophobic forces during gluten amyloid fibril formation. The ability to control AF structure through adjusting NaCl concentration levels, i.e., modulating the gluten network, holds promise for both industrial and clinical applications, potentially enabling the modification of glycemic response and/or digestibility. Future research should focus on enhancing the understanding of potential functional applications of AFs, such as texture modification, nutrient delivery, and colloidal stabilization in foods, alongside their potential health implications, to ensure their safe utilization in functional foods.

## Figures and Tables

**Table 1:** Effect of NaCl on the secondary structure distribution of AFs formed from noodles during the cooking process.

Salt Concentration/%	Cooking Time/min	β-Sheet/%	Random Coil/%	Alpha-Helix/%	β-Turning Angle/%
0	0	29.8 ± 0.07 ^Db^	13.6 ± 0.14 ^Aa^	18.0 ± 0.04 ^Aa^	38.6 ± 0.07 ^Aa^
	1	31.2 ± 0.14 ^Cb^	12.3 ± 0.12 ^Ab^	18.8 ± 0.03 ^Aa^	37.8 ± 0.07 ^ABa^
	3	31.5 ± 0.35 ^Cc^	11.1 ± 0.25 ^Ac^	19.7 ± 0.22 ^Aa^	37.8 ± 0.28 ^ABa^
	5	33.3 ± 0.57 ^Bb^	13.0 ± 3.21 ^Ab^	17.3 ± 2.56 ^Aa^	36.4 ± 0.07 ^ABb^
	7	37.2 ± 0.85 ^Ab^	11.3 ± 2.60 ^Ab^	20.8 ± 2.74 ^Aa^	30.8 ± 6.15 ^Bab^
0.4	0	36.6 ± 0.07 ^Da^	14.5 ± 0.05 ^Da^	15.8 ± 0.05 ^Ab^	33.1 ± 0.07 ^Aa^
	1	39.1 ± 0.00 ^Ca^	17.7 ± 0.00 ^Ca^	17.4 ± 0.00 ^Aa^	25.8 ± 0.00 ^Bb^
	3	39.5 ± 0.28 ^BCa^	17.7 ± 0.56 ^Ca^	20.3 ± 4.55 ^Aa^	22.5 ± 4.28 ^Bc^
	5	39.9 ± 0.41 ^Ba^	19.7 ± 0.59 ^Ba^	16.1 ± 1.39 ^Aa^	24.3 ± 0.41 ^Bc^
	7	41.5 ± 0.04 ^Aa^	21.8 ± 0.24 ^Aa^	14.7 ± 0.15 ^Ab^	22.0 ± 0.13 ^Bb^
1.6	0	27.8 ± 2.28 ^Cb^	16.2 ± 4.82 ^Aa^	12.3 ± 1.42 ^Cc^	39.4 ± 5.30 ^Aa^
	1	38.6 ± 1.41 ^Aa^	11.6 ± 1.29 ^Ab^	20.5 ± 0.27 ^ABa^	29.4 ± 0.42 ^Aab^
	3	34.0 ± 0.28 ^Bb^	12.6 ± 0.61 ^Ab^	23.2 ± 0.11 ^Aa^	30.2 ± 0.99 ^Ab^
	5	33.3 ± 0.57 ^Bb^	12.1 ± 0.77 ^Ab^	19.1 ± 0.53 ^Ba^	35.5 ± 0.78 ^Ab^
	7	33.2 ± 1.84 ^Bc^	12.9 ± 2.07 ^Ab^	21.1 ± 2.46 ^ABa^	32.8 ± 6.36 ^Aab^
2.0	0	28.9 ± 1.34 ^Bb^	13.1 ± 0.68 ^ABa^	18.1 ± 0.62 ^Aa^	39.9 ± 1.27 ^Aa^
	1	32.5 ± 1.48 ^Ab^	12.9 ± 1.84 ^ABb^	21.3 ± 2.91 ^Aa^	33.4 ± 6.22 ^Aab^
	3	30.0 ± 0.42 ^ABd^	12.3 ± 0.24 ^ABbc^	18.9 ± 0.19 ^Aa^	38.8 ± 0.42 ^Aa^
	5	30.7 ± 0.36 ^ABc^	11.0 ± 0.56 ^Bb^	20.0 ± 0.42 ^Aa^	38.2 ± 0.47 ^Aa^
	7	29.2 ± 0.35 ^Bd^	14.2 ± 0.31 ^Ab^	17.9 ± 0.03 ^Aab^	38.7 ± 0.07 ^Aa^

Note: Different capital letters indicate significant differences for the same salt concentration and different heating times; different lower-case letters indicate significant differences for different salt concentrations and the same heating times. Different superscript letters indicate significant differences (*p* < 0.05) according to Tukey’s HSD test.

**Table 2:** Effect of NaCl on the surface hydrophobicity of AFs formed from noodles during the cooking process.

Heating Time/min	0%	0.4%	1.6%	2.0%
0	1398 ± 193 ^cB^	3796 ± 366 ^cA^	1099 ± 282 ^bB^	1670 ± 321 ^cB^
1	6219 ± 32 ^bA^	4971 ± 248 ^cB^	6246 ± 468 ^aA^	4976 ± 676 ^bB^
3	6412 ± 208 ^bA^	6456 ± 223 ^bA^	6432 ± 534 ^aA^	5854 ± 381 ^abA^
5	6861 ± 398 ^bB^	8726 ± 536 ^aA^	5818 ± 377 ^aC^	6166 ± 341 ^aBC^
7	8553 ± 788 ^aA^	9980 ± 935 ^aA^	5472 ± 472 ^aB^	5923 ± 295 ^abB^

Note: Different lower-case letters indicate significant differences for the same salt concentration and different heating times; different capital letters indicate significant differences for different salt concentrations and the same heating times. Different superscript letters indicate significant differences (*p* < 0.05) according to Tukey’s HSD test.

## Data Availability

The original contributions presented in the study are included in the article; further inquiries can be directed to the corresponding author.

## Abbreviations

The following abbreviations are used in this manuscript:

AFs	Amyloid fibrils
NaCl	Sodium chloride
ThT	Thioflavin T
FTIR	Fourier transform infrared spectroscopy
AFM	Atomic force microscopy
SE-HPLC	Size exclusion high-performance liquid chromatography
H0	Surface hydrophobicity

## Appendix A

**Table A1:** The kinetic parameters of primary amyloid fibril formation.

Sample	A1(FU)	A2 (FU)	t1/2 (min)	Tc (min)	(*df/dt*) (FU min^−1^)	R2
0%	9882.48 ± 1997.81	34,312.42 ± 998.09	0.67 ± 0.06	0.19 ± 0.04	31,325.25 ± 8359.19	0.979
0.4%	11,572.59 ± 2183.15	39,193.17 ± 1187.69	0.72 ± 0.06	0.20 ± 0.05	35,171.12 ± 9823.06	0.978
1.6%	15,053.99 ±1597.24	40,474.09 ± 1552.08	0.77 ± 0.04	0.09 ± 0.03	67,541.97 ± 25539.60	0.95
2.0%	16,024.53 ± 2100.52	42,057.91 ± 2104.68	0.78 ± 0.05	0.08 ± 0.04	77,535.67 ± 40442.19	0.94
